# A Systematic Review of Physical Activity Intervention Programs in ASEAN Countries: Efficacy and Future Directions

**DOI:** 10.3390/ijerph19095357

**Published:** 2022-04-28

**Authors:** Yi-Shin Lee, Michael Chia, John Komar

**Affiliations:** Department of Physical Education and Sport Science, National Institute of Education, Nanyang Technological University, Singapore 637616, Singapore; nie21.lys3082@e.ntu.edu.sg (Y.-S.L.); michael.chia@nie.edu.sg (M.C.)

**Keywords:** intervention, physical activity guidelines, physical activity level, Southeast Asia, policymaking

## Abstract

A systematic review was conducted on the efficacy of interventions to improve physical activity. PubMed, Scopus and Web of Science were scanned for eligible studies published from 1978 to August 2021, resulting in a total of 52 relevant studies for review. The Downs and Black checklist was used as a quality assessment ool for a risk of bias assessment. The 52 studies were then broadly categorised into three major approach types: informational, behavioural and/or social, as well as direct. Within each major approach, studies were further sub-categorised into more specific intervention types before being assessed for their efficacy and applicability. Overall, the intervention types that seemed to be the most efficacious in increasing physical activity levels were those that involved home-based information provision, community-wide campaigns, incentivised change, individually adapted health behaviour programs, family-based social support interventions and the provision of self-monitoring tools. However, the results must be interpreted holistically, as many of the successful interventions included more than one approach type and success is likely contingent on effectively addressing several concurrent facets. The systematic review is registered on PROSPERO. Registration number: 282752.

## 1. Introduction

A lack of sufficient physical activity (PA) has been associated with an increased prevalence of noncommunicable diseases (NCD) [1,2]. Sufficient PA has been defined as at least 150–300 min of moderate-intensity PA, 75–150 min of vigorous-intensity PA, or an equivalent combination per week in healthy, adult populations [3]. PA refers to any bodily movement produced by skeletal muscles that result in energy expenditure above the resting metabolic rate, which may be unstructured and everyday life activity, an exercise that includes pre-arranged deliberate and repetitive activity, as well as grassroots sports and competitive sports [4].

Given how cardiovascular disease, hypertension and obesity are some of the leading health issues in Asia [5], and that Southeast Asia is projected to contribute to a quarter of 52 million annual NCD-related deaths by 2030 [6] while concurrently possessing a lower prevalence of sufficient PA compared to the global average [7], it is essential that these concerns are addressed through effective policymaking aimed at raising PA levels.

However, it is important to note that while there has been previous work examining the efficacy of PA interventions in other countries or regions [8,9,10], these may not necessarily work in Southeast Asia due to potential cultural differences and motivations for PA participation [11,12,13]. For example, when compared across cultures, gender may be a potential predictor of PA participation [11]. It may also be particularly pertinent that PA is less culturally acceptable amongst women in such cultures [12] and that several countries within the region have notable proportions of Muslims (e.g., Indonesia, Brunei, Malaysia, Singapore) that may require a different approach to influencing PA participation [13]. Thus, this review will serve to focus specifically on reviewing the efficacy of PA interventions conducted within the Association of Southeast Asian Nations (ASEAN), which includes Brunei, Cambodia, Indonesia, Laos, Malaysia, Myanmar, the Philippines, Singapore, Thailand and Vietnam.

This review will categorise the various interventions described in literature into different categories and evaluate the efficacy of those categories to formulate an idea of an optimal intervention type. Based on the findings, several considerations for future directions will be suggested for research and policymaking with the goal of increasing PA levels in the member states within ASEAN.

## 2. Materials and Methods

### 2.1. Search Process and Inclusion Criteria

Following the results of an earlier paper [14] by the same authors, we wanted to further evaluate the efficacy of the *experimental studies*, in particular. This includes both randomised controlled trials and quasi-experimental study designs; essentially, any study with an *intervention* aimed at affecting PA levels. To note, while RCTs may be widely regarded as the gold standard for experimental designs [15], it is not often the case that this is the most plausible study design to employ in healthcare settings due to possible ethical [16] or practical concerns, e.g., there is a risk of contamination bias when study participants are within very close geographical and/or social proximity. Thus, we have included quasi-experimental designs in our review as well.

Specifically, the PICO for this review is as follows:

(a) P: Individuals of any age; males and females; from ASEAN countries;
(b) I: Any type of intervention that is aimed at influencing PA levels, regardless of whether the intervention is aimed at influencing it as a primary or secondary variable;
(c) C: Placebo;
(d) O: PA level.

Studies examined in this review were gathered according to the preferred reporting items for systematic reviews and meta-analyses (PRISMA) guidelines. Three electronic databases (i.e., PubMed, Scopus and Web of Science) were searched; from 1998 (when the first paper relevant to the review was published) until 31 August 2021, to identify articles containing the following keywords and combinations:

(a) Physical Activity; AND
(b) Indonesia OR Philippines OR Malaysia OR Singapore OR Laos OR Brunei OR Myanmar OR Burma OR Thailand OR Cambodia OR Vietnam OR ASEAN OR Southeast Asia; AND
(c) Measurement OR Method OR Survey OR Consensus.

The 52 included studies carried out interventions and measured the PA level change. These studies were published in the English language between 1978 and August 2021. There were initially 1251 articles found on PubMed, 1284 in Scopus and 550 on Web of Science. Rayyan QCRI was used to compile the results and manually resolve the duplicates, resulting in 1213 unique studies. After screening the abstracts for the relevant information, 44 reports were determined to have met the eligibility criteria and were sought for retrieval.

**Figure 1:**
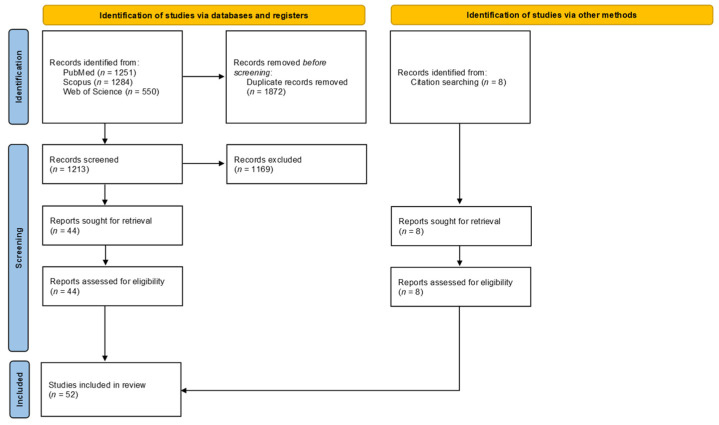
PRISMA inclusion/exclusion process for the systematic review.

Studies were excluded if they were inaccessible, irrelevant, did not include an intervention, did not include a measurement of PA level change, were conducted in non-ASEAN populations, the PA data between ASEAN and non-ASEAN countries was indiscernible, and were not written in the English language. All 44 reports were retrieved. An additional 8 articles were added through other sources (i.e., through citation searching) for a total of 52 articles included in this review. The PRISMA inclusion/exclusion process [17] is illustrated in Figure 1.

### 2.2. Quality Assessment and Level of Evidence

The Downs and Black checklist was used to assess the risk of bias. It includes 27 items that measure the quality of the reporting, the external validity, the internal validity (bias and confounding) and the power of a study [18]. A modified version was used here [19] as the information regarding the different levels of power stipulated in the original checklist is often difficult to find. Randomised controlled trials (RCT) would have a maximum possible score of 28, while non-randomised trials would have a maximum possible score of 25. The following scores corresponded with the following quality levels: excellent (26–28); good (20–25); fair (15–19); and poor (≤14). Thus, only RCTs would be able to attain an excellent score. Further, the studies were assigned the following study levels based on the Oxford Centre for Evidence-based Medicine (OCEBM) [20] to assess the level of evidence of the benefits demonstrated by the various interventions: RCTs (Level 2); and non-randomised controlled cohort/follow-up studies (Level 3). However, numerical quality assessment scales such as the Downs and Black checklist may potentially be weaker at identifying studies with an increased risk of bias when only the summary scores are taken into consideration [21]. To this effect, the OCEBM levels of evidence and Downs and Black checklist sub-scores of each study have been provided in Table 1, allowing for a more holistic approach toward assessing the various sub-domains of study quality.

The average Downs and Black checklist score for reporting was 7.8 (1.1) points out of 10, suggesting that a majority of the studies included in the review had generally good reporting practices. The mean external validity score was 1.3 (0.7) out of 3, suggesting that most studies included in the review may have poor generalisability to other settings. The mean internal validity score (bias) was 4.5 (1.0) out of 7, suggesting that most studies may have a fair amount of internal validity pertaining to any bias concerning the measurement of the intervention and PA levels. The mean internal validity score (confounding) was 3.1 (1.5) out of 6, suggesting that most studies may have a fair amount of internal validity pertaining to any type of selection bias that may have been present. The mean power score was 0.5 (0.5) out of 1; approximately half of the studies did not include some type of power calculation, which may have resulted in some of the studies being underpowered and unable to detect the effects they were looking for.

We would like to emphasise that there were a variety of methodologies used for these studies. Beyond the different study designs, there was also the use of different types of both objective and subjective measurement tools as well. For a better idea of how these may affect a study and its findings, please refer to an earlier paper we wrote [14]. These should be taken into consideration along with the OCEBM and risk of bias tools that have been used in this review.

### 2.3. Selection of Outcomes for Review

A fair number of the studies included in this review measured primary outcomes other than changes in PA levels, with some of the studies not measuring PA as a primary outcome (i.e., their interventions were not directed specifically towards affecting PA levels). For example, nutritional interventions that measured PA as a secondary outcome. However, these studies were still included as there may be relationships that may emerge from the analysis of these studies and shed light on how future interventions may be structured to best increase PA levels. PA levels were measured in a variety of ways, including, but not limited to, weekly step averages, the prevalence of low PA and minutes of PA.

### 2.4. Determination of Approach Categories

Previous work has established a useful framework for categorising PA interventions and has been adapted for this review [9]. However, instead of the last category of ‘environmental and policy approach’ as defined in that review, we have categorised the third approach as ‘direct’ due to the lack of studies that utilised the policy changes. Thus, the three categories are: (a) informational, (b) behavioural and/or social and (c) direct approaches. Informational approaches focus on promoting PA through the provision of educational material, behavioural and/or social approaches focus on promoting PA by teaching behavioural management skills and/or altering the social environment available to the individual, while direct approaches focus on promoting PA by directly offering increased opportunities to perform PA.

## 3. Results

The following sections categorise the findings into approaches as per outlined in Section 2.4. For a breakdown of study characteristics, interventions and findings for all studies included in the review, please refer to Table 2.

### 3.1. Informational Approaches

Informational approaches generally encompass the provision of educational materials, in the form of brochures, lectures, classes, etc., that may help increase PA levels. For example, the reviewed studies included educational material, such as education sessions on basic diabetes self-management principles (that include increasing PA), education sessions on hypertension self-management, nutrition education sessions and material on how exercises are performed. The educational material is provided with the intent of increasing awareness around the benefits of PA or the modification of certain behavioural risk factors on disease outcomes and lowering the barriers to exercise engagement. The approaches reviewed here are broadly classified into (a) school-based (b) hospital-based (c) home-based (d) community-wide and (e) point-of-decision prompts interventions aimed at increasing information provision. A total of 21 out of the 52 studies included for review utilised an informational approach to affecting PA levels. Of the 21 studies, 14 studies provided evidence for the informational approach to increasing PA levels, while the remaining 7 studies did not.

#### 3.1.1. School-Based Informational Provision

School-based interventions generally involve information provision largely within a school or classroom setting, whether the delivery medium is through physical lectures or technology. There were six studies that utilised this [22,23,24,25,26,27].

##### Efficacy

Of the six studies that utilised school-based information provision, three provided evidence for the efficacy of the intervention type in increasing PA levels [23,25,27]. This might be explained by the broader coverage of the intervention detailed in [23] as it included teacher and parental involvement in addition to the educational classes, addressing aspects of social support as well. With the other two successful interventions [25,27], while successful, the interventions involved only the regular dissemination of *nutrition-related* information. The concurrent increase in PA might possibly be explained by participants developing better lifestyle habits in general, i.e., improving PA habits due to the indirect positive influence of improving nutritional status [28]. Whereas [22] only involved displaying exercise videos to the students. Furthermore, while [26] utilised a nutrition intervention similar to [25], using the Malaysian dietary guidelines, only the latter was successful in increasing PA levels. A potential explanation could be the difference between the baseline PA measures for both studies, i.e., the standard deviation for the baseline measures in [26] was highly dispersed while those in [25] were much smaller, potentially leading to a non-significant finding after statistical analysis in the former. While [24] utilised a multicomponent healthy lifestyle program that targeted several facets of the participants’ lifestyle, it was unsuccessful in increasing PA levels.

##### Applicability

While the current evidence is ambivalent regarding the efficacy of such an intervention type in increasing PA, the evidence here suggests that school-based information provision is a potentially effective method for increasing PA.

#### 3.1.2. Hospital-Based Informational Provision

Hospital-based interventions generally involve information provision largely within a hospital setting. There was one study that utilised this [29].

##### Efficacy

Of the one study that utilised hospital-based information provision, it provided evidence for the efficacy of the intervention type in increasing PA levels.

##### Applicability

While the sample size is too small to draw a definitive conclusion, the evidence here suggests that hospital-based information provision is a potentially effective method of increasing PA.

#### 3.1.3. Home-Based Informational Provision

Home-based interventions differ from both school-based and hospital-based interventions in the way the majority of the information is delivered or consumed. While the information may be provided by individuals operating out of schools or hospitals, the participants received or consumed the majority of this information at home. This is an important distinction, as there is an immediate lack of authority that may otherwise influence the way in which the participants behave in response to the information [30], unlike in school-based and hospital-based interventions. The interventions included providing myriad PA-related information, such as through the dissemination of exercise videos, provision of self-management material for metabolic diseases and increasing knowledge of available facilities for PA. There were nine studies that utilised this intervention type [31,32,33,34,35,36,37,38,39].

##### Efficacy

Of the nine studies that utilised home-based information provision, eight provided evidence for the efficacy of the intervention type in increasing PA levels [31,32,33,34,35,36,37,38], while one did not [39]. Of the eight studies that were successful in increasing PA levels, the interventions included the dissemination of exercise videos [31], a multifactorial falls prevention program [32], self-management material for metabolic diseases [33,38], a combination of PA and dietary guidelines [34,35], and a provision of knowledge on structured outdoor activities [36,37]. Pertaining to the study that was not successful in increasing PA, the intervention involved text messaging that provided instructions on exercise and praise for positive behaviour [39]. Importantly, many of the successful interventions that have been listed here involved a variety of other intervention types in addition to information provision. As detailed above, many interventions involved a more holistic set of information that revolved around managing various aspects of one’s lifestyle, extending beyond just an increase in PA levels, such as the management of metabolic disease parameters, dietary guidelines and eye care habits. Certain studies included some form of familial support as well [36,37]. It is thus unsurprising that, for the one study that was unsuccessful, the intervention was relatively limited in scope.

##### Applicability

There is a large amount of evidence pointing toward the efficacy of home-based interventions in increasing PA levels, potentially making this a viable intervention style to pursue in future studies or when considering PA-related policymaking. However, it must be noted that for the overwhelming majority of the studies that were successful, there was some form of an additional component to the intervention, whether it was information provided on other aspects of lifestyle or social support.

#### 3.1.4. Community-Wide Campaigns

Community-wide campaigns involve multiple, highly visible interventions that involved many members, beyond the participants themselves, in a community across various sectors. There were four interventions in this review [40,41,42,43] that utilised a community-wide campaign.

##### Efficacy

Of the four studies that utilised a community-wide campaign approach, three provided evidence for an increase in PA levels [40,41,43], while one did not [42]. Of the studies that were successful, one study provided education, increased access to exercise equipment and social support in the form of walking groups [40], another provided group education sessions with additional individual counselling sessions aimed at behaviour change [41] while the third study involved the participants engaging in myriad activities aimed at both cognitive and physical stimulation [43]. With regards to the study that was unsuccessful in increasing PA levels, there was in fact a significant increase in physical *inactivity* levels after the 3-year intervention. However, there are several issues with drawing a firm conclusion about the effects of this intervention on affecting PA levels—including that the intervention’s purpose was not limited solely to increasing PA levels, the other interventions may have had a role in affecting PA levels and there may have been confounding factors due to the normal passage of time, given the long duration of the study, which may have affected PA levels regardless of the intervention.

##### Applicability

The current body of evidence suggests that community-wide campaigns are potentially an effective method for increasing PA levels. However, note that this intervention type tends to be more holistic in nature, going beyond mere information provision and often targeting various aspects of behaviour change as well.

#### 3.1.5. Point-of-Decision Prompts

Point-of-decision prompts are usually signs that are placed near stairs or elevators to encourage individuals to utilise the stairs. The intention is to provide information on a healthier alternative to an established behavioural norm [44]. There was only one intervention in this study that utilised point-of-decision prompts [45].

##### Efficacy

Of the one study in our review that utilised point-of-decision prompts, it must be noted that the study also compared its efficacy to another intervention that involved providing a weekly 1-h aerobics class. The chosen PA measure was steps in a day. The study provided no evidence that point-of-decision prompts were effective in eliciting an increase in PA, while the other intervention involving an aerobics class did.

##### Applicability

Applicability cannot be measured if effectiveness was not established.

### 3.2. Behavioural and Social Approaches

Behavioural and social approaches attempt to affect PA levels by teaching or creating incentive structures to encourage behavioural management skills and/or influencing changes in a person’s social support structure to affect behavioural change. These types of interventions usually encompass counselling sessions for individuals or groups. These approaches include but are not limited to teaching participants self-regulation techniques or techniques to alter self-efficacy with regards to engaging in PA and peer support groups. The approaches reviewed here are broadly classified into (a) peer support, (b) incentivised change, (c) self-regulation strategy, (d) individually adapted health behaviour change program, (e) family-based social support, (f) workplace-based social support, (g) healthcare provider training and (h) self-monitoring tools. A total of 23 out of the 52 studies included for review utilised a behavioural or social approach to affecting PA levels. Of the 23 studies, 16 studies provided evidence for the behavioural or social approach to increasing PA levels, while the remaining 7 studies did not.

#### 3.2.1. Peer Support

Peer support interventions are generally conducted in group settings, where participants may engage in a variety of activities, such as group counselling sessions, peer-led training programs and accountability in front of a group. There were four studies that utilised this [46,47,48,49].

##### Efficacy

Of the four studies that utilised peer support, one provided evidence for the efficacy of the intervention type in increasing PA levels [48], while the remaining three did not [46,47,49]. Of the one study that was successful in increasing PA levels, the intervention involved monthly meetings where the group would convene and go through their PA log diaries and were provided with motivation to further increase PA. Of the three studies that were unsuccessful in increasing PA levels, one was labelled as such due to the absence of significance testing [47]. With regards to the other two interventions, one involved group lifestyle counselling sessions [46] while another involved training peer leaders before getting them to lead whole and small group peer support programs [49].

##### Applicability

While the sample size is too small to draw a definitive conclusion, the evidence here suggests that peer support is a potentially effective method for increasing PA.

#### 3.2.2. Incentivised Change

Incentivised change interventions are not just interventions that provide some form of financial incentive for the participants, but interventions that specifically investigate the effect of an incentive or different types of incentives on affecting PA levels. There were three studies that utilised this intervention type [50,51,52].

##### Efficacy

Of the three studies that utilised incentivised change, all three were successful in increasing PA. In the study [50] that utilised the incentivised change intervention type, there was a comparison between 4 groups; a control group, a group only given a Fitbit pedometer (Fitbit-only), a group given a Fitbit pedometer and whose progress contributed to financial compensation towards a charity organisation (Charity), and another group given a Fitbit pedometer and whose progress contributed to financial compensation towards the participant (Cash). After 12 months, both the Fitbit-only and Charity groups displayed a significantly higher moderate to vigorous PA (MVPA) level than the control group, while the Cash group had a significantly lower MVPA level than the Fitbit-only group. However, in terms of mean daily steps, it was the Fitbit-only and Cash groups that were significantly higher than the control group. Of note, the incentives were given based on a certain number of steps being reached and not MVPA time. In essence, at least when comparing the efficacy of financial incentives, only financial compensation towards the participants themselves was successful in increasing PA levels. In [51], the groups that were given incentives demonstrated greater levels of PA. Further, [52] demonstrated the efficacy of providing financial incentives in increasing the daily step count.

##### Applicability

The current body of evidence suggests that the provision of financial incentives is effective in increasing PA levels. More importantly, [50] possibly further provides evidence that suggests financial incentives are only effective insofar as the compensation is directed towards the participants themselves, similar to the other two studies reviewed.

#### 3.2.3. Self-Regulation Strategy

Self-regulation strategy interventions attempt to teach participants methods of self-regulation to affect PA levels. Self-regulation involves attempts by an individual to alter one’s responses and inner states [53]. There was one study that utilised this intervention type [54].

##### Efficacy

Of the one study that utilised this intervention type, it was successful in increasing PA levels, but only in the men within the sample. The success of self-regulation strategies in increasing PA levels is corroborated in other literature for both men and women [55,56]. While there was no significant finding pertaining to PA levels in women, there was a non-significant increasing trend. A larger sample size may have potentially been able to detect a significant finding.

##### Applicability

While the sample size is too small to draw a definitive conclusion, the evidence here and in the previous literature suggests that self-regulation is potentially an effective strategy for increasing PA levels.

#### 3.2.4. Individually Adapted Health Behaviour Change Program

Individually adapted health behaviour change programs are interventions that attempt to alter participants’ health-related behaviour through teaching behaviour management skills, often based on established health behaviour change models [57,58], to affect PA levels. There were four studies that utilised this intervention type [59,60,61,62].

##### Efficacy

Of the four studies that utilised this intervention type, three were successful in increasing PA levels [60,61,62], while one was not [59]. Of the three successful interventions, all three utilised some form of combined intervention, with [60] incorporating elements of peer support, and [61,62] incorporating the direct provision of physical training components. Of note, [60] had two intervention groups, one utilising personalised feedback, and another utilising peer support in addition to the personalised feedback. Only the group with additional peer support had a significant increase in PA levels. Regarding the study that was unsuccessful in increasing PA levels, a possible reason could be that the intervention only provided nutrition counselling, which might have been insufficient in affecting PA levels.

##### Applicability

The sample of studies here points toward the efficacy of individually adapted health behaviour change programs in increasing PA levels. However, it must be noted that all of the studies that were successful in increasing PA levels also included components other than the individually adapted health behaviour change programs.

#### 3.2.5. Family-Based Social Support

Family-based social support interventions attempt to affect participants’ PA levels through the alteration of the familial aspect of their social support structure. There were three studies that utilised this intervention type [63,64,65].

##### Efficacy

Of the three studies that utilised this intervention type, all three were successful in increasing PA levels [63,64,65]. Of the three successful interventions, one targeted the mothers and sought to provide practical guidelines on childhood obesity management [63], another entailed the parents receiving nutritional education to manage their children’s diet and providing oral nutritional supplements to the children [64], while [65] was also a parent-centric intervention that sought to educate parents on various behavioural strategies aimed at preventing childhood obesity.

##### Applicability

The sample of studies here points toward the efficacy of family-based social support interventions in increasing PA levels. However, it is important to note that the commonality across the interventions that worked was that they were parent-centric interventions directed toward improving their child’s health outcomes. It may be easier to affect the PA levels of a child under the care of their parent since the latter is able to more strictly control the various lifestyle factors around their child.

#### 3.2.6. Workplace-Based Social Support

Workplace-based social support interventions attempt to affect participants’ PA levels through the alteration of aspects within their workplace. There were two studies that utilised this intervention type [66,67].

##### Efficacy

Of the two studies that utilised this intervention type, one was successful in increasing PA levels [66], while the other was not [67]. For the intervention that was successful, participants were provided with fitness trackers and involved in sedentary behaviour-reducing activities, while their physical and social environments were adjusted to be more conducive to that. The study that was not successful in affecting PA levels [67] was a workplace health promotion intervention that sought to alter several arms of social support related to the participants.

##### Applicability

The sample size is too small and the current evidence is ambivalent. Further research in the area is required to ascertain the efficacy of such an intervention on increasing PA levels.

#### 3.2.7. Healthcare Provider Training

Healthcare provider training interventions attempt to affect participants’ PA levels by teaching healthcare providers, who would come into contact with the participants, a variety of skills aimed at improving several aspects of patient care. There was one study that utilised this intervention type [68].

##### Efficacy

The study that utilised this intervention type was not successful in increasing PA levels. However, of note, the healthcare provider training did not focus solely on improving PA and the related portion of the intervention was mostly about training nurses in motivational conversation to set priorities for self-care and medication adherence.

##### Applicability

Applicability cannot be determined if there was no evidence of effectiveness.

#### 3.2.8. Self-Monitoring Tools

Self-monitoring tool interventions attempt to affect participants’ PA levels by providing certain tools that may assist in self-monitoring and thus, encouraging PA behaviour. There is some evidence that demonstrates the efficacy of providing participants with self-monitoring tools in increasing motivation to participate in PA, enhancing self-monitoring skills and thus, greater awareness around goal behaviour discrepancy to improve their lifestyles [69,70]. There were four studies that utilised this intervention type [71,72,73,74].

##### Efficacy

Of the three studies that were successful in using this intervention type, one required the intervention group to use a mobile application for 6 months to track weight twice weekly, their diet and PA daily and to communicate regularly with the research dietitians through the application [72]. Another involved providing the intervention participants with face-to-face counselling on PA, as well as filling out a park-based PA prescription sheet with an outlined goal they had committed to [73]. Finally, the third study that was effective in increasing PA levels using this intervention type provided participants with pedometers to track and provide feedback on their daily step count [74]. While the last study [71] mentioned an increase in the number of weekly recorded pedometer steps from 59,560 to 87,286 after seven weeks of their intervention, there was no significant testing and thus the study was marked as not being effective However, given the study’s results, the success of the intervention by [74] and the similarities across the two interventions, there is reason to believe that the study would have been found to be effective if statistical testing were done.

##### Applicability

The current body of evidence and previous literature suggests that the use of self-monitoring tools is an effective intervention type to increase PA levels.

### 3.3. Direct Approaches

Of the eight studies that adopted direct approaches, four provided exercise sessions [75,76,77,78], one provided exercise equipment [79], two provided oral nutritional supplementation [80,81] and the last provided medication [82].

#### 3.3.1. Efficacy

Four of the eight studies were successful in increasing PA levels. Of the four studies that were successful in increasing PA levels, one provided exercise classes [76], one provided increased time to play [78], one provided oral nutritional supplementation [81] and the last provided medication [82]. The remaining four were not successful [75,77,79,80].

#### 3.3.2. Applicability

There seems to be ambivalence regarding the efficacy of such an intervention type on increasing PA levels. There is insufficient evidence to determine the efficacy of direct approaches to increasing PA levels.

## 4. Discussion

Upon reviewing the articles, it was found that 66.7% of interventions that utilised an informational approach, 69.6% of interventions that utilised a behavioural and/or social approach, and 50% of interventions that utilised a direct approach were successful in increasing PA levels, suggesting a greater efficacy for informational and behavioural and/or social approaches. However, more importantly, many of the interventions that were successful were those that adopted a multi-pronged approach to influencing PA levels instead of just utilising a single approach. The following will discuss these in detail as well as lay out potential future steps to consider.

### 4.1. Overall Efficacy of Reviewed Interventions

Of the intervention types reviewed, the categories with a sufficient amount of evidence demonstrating efficacy in increasing PA levels include home-based information provision [31,32,33,34,35,36,37,38,39], community-wide campaigns [40,41,42,43], incentivised change [50,51,52], individually adapted health behaviour programs [59,60,61,62], family-based social support interventions [63,64,65] and the provision of self-monitoring tools [71,72,73,74]. However, that does not mean that the other intervention types are necessarily ineffective in increasing PA levels, but that more research may need to be done to ascertain their effect on PA levels. Further, it is worth noting that many of the interventions that were successful in increasing PA levels utilised a combination of intervention types, most commonly some form of information provision and social support. Future national policies that are aimed at increasing PA levels should therefore consider focusing on multicomponent strategies that incorporate elements of information provision and social support. Speaking more broadly, a vision of PA within such societies would therefore be one where interventions to influence PA would not be limited to a single section of an individual’s life and instead permeate the various facets of it (i.e., in the form of environmental changes melded with information provision and social support). For example, this might be in the form of nationwide efforts to increase PA through active commuting by the conversion of car-friendly roads to active commuting-friendly roads that allow safe bicycling, while distributing pamphlets on the benefits of such activity through community centres and hospitals that could be then taken back home for parents to educate their children with.

### 4.2. The Need to Account for Local Cultural Considerations in PA Promotion Programs

An important aspect for consideration in the promotion of PA in ASEAN is the myriad of potential cultural limitations that may exist. For instance, approximately 42% of the combined population across the ASEAN countries are Muslim [83] and depending on how the religion is practised, there may be additional factors to consider when promoting PA in these societies [13,84,85]. Specifically, the religion itself promotes PA [86] but there may be differences in the ways in which it can be facilitated given certain restrictions within the society, such as stipulations on garb and intermingling between individuals of the opposite sex. Of course, the major religions in ASEAN countries go far beyond that of just Islam. However, it seems to present the most unique challenges to PA promotion and, thus, our decision to focus on this as an example.

As another example, for a country like Myanmar, which has a female labour force of 46% of its population [87], a workplace intervention may not be the most optimal way to broadly target an increase in female PA participation in the country.

Hence, while this review has identified several intervention types that may effectively increase PA levels, the efficacy may be influenced by the cultural contexts in which they are based. Accordingly, policymaking bodies using this information should also consider how different intervention types could, or should not, be used based on other coinciding factors.

### 4.3. Towards a Potential Addendum for Local PA Guidelines

**Figure 2:**
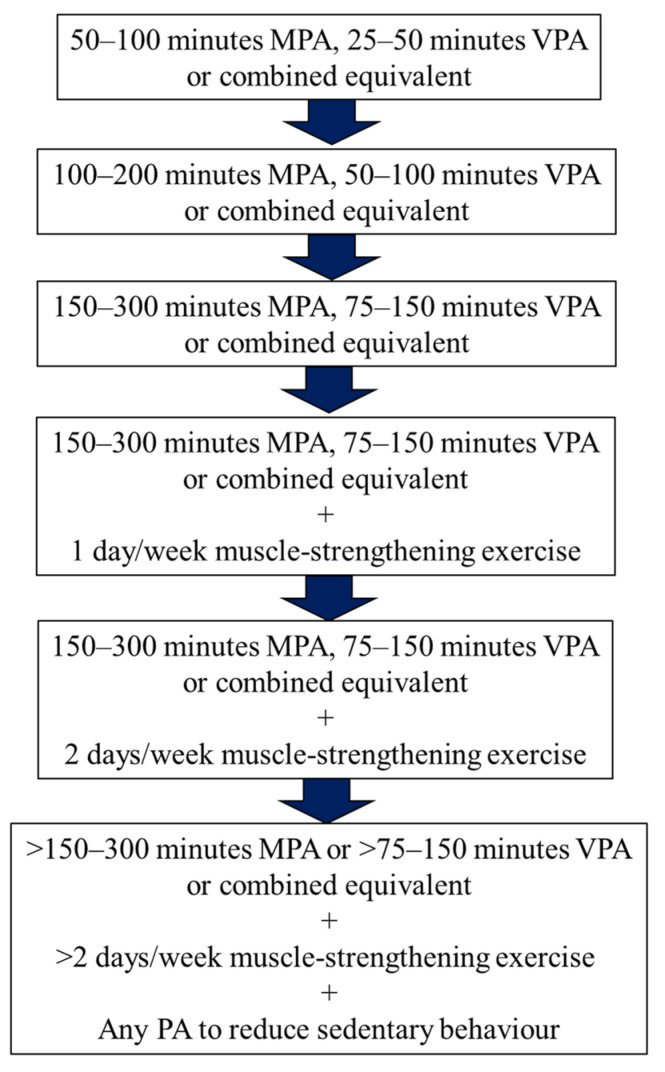
Chart demonstrating an example of intermediary steps that can be taken to achieve optimal physical activity goals, as defined by the World Health Organization.

As pointed out in the previous literature, the mere existence of PA guidelines is likely insufficient in effecting change [3] in the PA levels of a population. The same article emphasised the importance of how this information should be communicated and how policies should be implemented to maximise the impact on PA levels. To add to this, it has been shown that commitment declines as a goal either becomes more difficult or is perceived as being more difficult to achieve [88]. For an unfit individual who does not habitually engage in PA, a jump to a goal of 150–300 min of moderate-intensity or 75–150 min of vigorous-intensity PA may be perceivably difficult and potentially cause these individuals to experience reduced goal commitment. Hence, while the World Health Organization (WHO) recognises that all PA in daily life is beneficial [89], it may be prudent if more could be done to establish intermediary steps to encourage goal commitment, to ultimately reap the optimal health benefits associated with reaching the targeted PA levels. An example of such a structure is given in Figure 2. An addendum like this may provide some additional structure for healthcare practitioners to better prescribe progressive increases towards the optimal range.

Further, there are many studies that currently classify participants into “in/active” or “having/not met PA guidelines”, which may lack precision in assessing both the current state and progress that participants may have made in a study with regards to achieving sufficient PA levels. For instance, a participant may have gone from minimal amounts of PA to a level just shy of being classified as “active” or “having met PA guidelines” and they would still be considered as not having made any progress due to the dichotomous categorisation. The aforementioned addendum may, therefore, create higher precision in assessing the effect of an intervention on PA levels by establishing standardised, intermediary benchmarks.

## 5. Strengths and Limitations

The strength of this review is in its ability to provide a different cultural perspective—individuals from different cultures may have distinct reasons for participating in PA and thus respond better to certain intervention types. On the other hand, a limitation of this review is that many of the studies included are non-randomised controlled trials or single-arm studies, which may affect the quality of the findings due to the lack of control. However, as discussed earlier, this is unavoidable in many experimental designs within healthcare. Importantly, this is where the risk of bias assessments is crucial and we encourage readers to scrutinise the findings of the studies within the context established (i.e., using the OCEBM levels and Downs and Black checklist scores we have provided). Further, there were potentially studies that we were unable to include due to our inability to understand the language. If possible, researchers in this field may want to explore the translation of such texts for inclusion in future reviews.

## 6. Conclusions

Broadly speaking, the review found that interventions that utilised an informational or behavioural and/or social approach were more successful in increasing PA levels, suggesting a greater efficacy for these approaches. However, these results need to be interpreted holistically, as many successful interventions are a combination of intervention types. Further, future policymaking decisions regarding PA interventions should be considered within the potential limits of cultural constraints. It may also be worth considering the establishment of standardised intermediary benchmarks for the optimal ranges of PA goals set by WHO.

## Figures and Tables

**Table 1:** Summary of OCEBM level, Downs and Black checklist sub-scores.

Article	OCEBM Level ^1^	R ^2^	EV ^3^	IV:B ^4^	IV:C ^5^	P ^6^	Total ^7^
Bilger et al., 2021	2	8	3	5	6	0	22
Chew et al., 2021	3	7	2	5	3	1	18
Juniarti et al., 2021	2	8	3	5	4	1	21
Lim et al., 2021	2	8	3	5	6	1	23
Maphonga et al., 2021	3	7	2	5	1	1	16
Petrunoff et al., 2021	2	8	3	5	4	0	20
Saad et al., 2021	3	8	3	5	1	1	18
Teo et al., 2021	3	7	3	5	1	1	17
Hidrus et al., 2020	2	9	1	7	4	0	21
Cheah et al., 2019	3	8	1	4	2	0	15
Nik Yahya et al., 2019	3	7	1	3	2	0	13
Pengpid et al., 2019	2	7	1	3	4	0	15
Pichayapinyo et al., 2019	3	7	1	3	2	0	13
Rizal et al., 2019	3	8	1	5	2	0	16
Zulkifi et al., 2019	3	6	1	3	2	0	12
Liau et al., 2018	3	9	1	3	3	0	16
Mok et al., 2018	3	8	1	4	2	1	16
Nguyen et al., 2018	3	9	2	5	0	0	16
Nur Emilia et al., 2018	2	9	1	4	3	1	18
Omar et al., 2018	3	8	1	3	1	0	13
Suttanon et al., 2018	2	9	1	7	5	1	23
Widyastuti et al., 2018	2	9	1	5	4	1	20
Yatim et al., 2018	3	7	1	4	2	1	15
Chawla et al., 2017	3	8	1	4	2	0	15
Huei et al., 2017	2	8	1	4	3	0	16
Juliana et al., 2017	2	8	1	4	4	1	18
Karintrakul et al., 2017	2	8	1	3	4	0	16
Sanee et al., 2017	3	7	1	4	0	1	13
Tran et al., 2017	2	7	1	4	3	0	15
Zahtamal et al., 2017	3	7	1	4	2	0	14
Finkelstein et al., 2016	2	6	1	6	5	1	19
Haya et al., 2016	3	8	1	4	3	1	17
Ibrahim et al., 2016	3	9	1	4	3	1	18
Jafar et al., 2016	3	9	1	5	2	0	17
Müller-Riemenschneider et al., 2016	2	9	2	6	5	1	23
Nhung et al., 2016	2	7	1	5	3	0	16
Ethisan et al., 2015	3	8	2	4	3	0	17
Huynh et al., 2015	3	8	1	5	3	0	17
Ng et al., 2015	2	8	1	6	6	1	22
Sazlina et al., 2015	2	10	1	6	6	1	24
Ngo et al., 2014	2	8	1	5	5	0	19
Sriramatr et al., 2014	2	6	1	4	5	1	17
Suwanpasu et al., 2014	2	6	1	3	3	1	14
Finkelstein et al., 2013	2	9	1	6	5	0	21
Lua et al., 2013	2	9	1	3	4	1	18
Soon et al., 2013	2	9	1	4	2	0	16
Nguyen et al., 2012	3	7	2	4	1	0	14
Tan et al., 2011	2	8	1	5	3	1	18
Wafa et al., 2011	2	7	1	5	3	1	17
Chia, 2009	3	3	1	5	2	0	11
Muda et al., 2006	2	8	1	4	3	1	17
Hadju et al., 1998	2	8	0	5	2	0	15

^1^ OCEBM level of evidence, ^2^ Downs and Black (D&B) reporting score, ^3^ D&B external validity score, ^4^ D&B internal validity: bias score, ^5^ D&B internal validity: confounding (selection bias) score, ^6^ D&B power score, ^7^ Total D&B score.

**Table 2:** Breakdown of study characteristics, interventions and findings for all studies included in the review.

Paper	Population ^1^	Intervention	Results ^2^
Bilger et al., 2021	*n* = 240 Singapore Adults M/F Type 2 diabetes	All participants received usual care. There was one control group, one process-based incentive group, and one outcome-based incentive group. The process-based incentive participants earned financial incentives contingent on meeting specified intermediary health behaviours. The outcome-based incentive participants earned financial incentives contingent on meeting certain health behaviour outcomes. The intervention ran for 6 months.	Incentive groups’ mean number of physically active days during the last week of intervention was higher compared to the control group (2.35 vs. 1.24).
Chew et al., 2021	*n* = 129,677 to 690,233 Singapore Adults M/F	Participants were given fitness trackers. Financial incentives were given to participants for hitting certain daily step goals. There were 3 waves of varying durations.	For all 3 waves, there was an increase in mean daily steps from baseline to the end of the intervention. Wave 1 (4512 to 8675), Wave 2 (6221 to 8463) and Wave 3 (7432 to 9077).
Juniarti et al., 2021	*n* = 90 Indonesia Older adults M/F	Intervention group participants engaged in reading activities, listening, writing, drawing and exercise activities across a span of 4 weeks. Sessions were led by community volunteers.	The intervention group increased PA from 18.56 to 19.71 (PASE), while the control group did not increase PA.
Lim et al., 2021	*n* = 204 Singapore Adults; older adults M/F	Participants assigned to the intervention group were required to use the app for 6 months to track weight twice weekly and diet and physical activity daily, and to communicate regularly with the research dietitians via the app. Intervention participants chose a weight loss goal and were encouraged to achieve both nutritional and PA goals.	Change in PA (min/wk) was higher in the intervention group compared to the control group by 53.4 and 62.4 at the 3-month and 6-month marks, respectively.
Maphong et al., 2021	*n* = 78 Thailand Adults M/F	Participants in the intervention group were involved in sedentary behaviour-reducing activities over 8 weeks. At the individual level, participants were given information and at the organisational level, the physical and social environments of the organisation were adjusted.	Increase in METs for the intervention group (1.03 to 1.17), while there was no change for the control group.
Petrunoff et al., 2021	*n* = 160 Singapore Adults M/F	Participants in the intervention group received face-to-face counselling on PA, during which they also completed a park prescription sheet with a trained study team member for 26 weeks. The prescription sheet outlined a goal they committed to specifying the frequency, intensity, time and location of exercise in parks. Participants subsequently received a sheet to plan their weekly park PA and information brochures about parks in their neighbourhood.	The amount of park PA (min/month) had a significant mediating effect on recreational MVPA (min/wk) at 26.50 [6.65, 49.37].
Saad et al., 2021	*n* = 128 Malaysia Older adults M/F	Participants in the intervention group were given pedometers for daily feedback for the 12-week intervention.	Number of daily steps increased from 3403 to 6975 for the intervention group, while there was no significant change in the control group.
Teo et al., 2021	*n* = 582 Malaysia Children M/F	The 3-month intervention consisted of delivering nutrition education to the children through their teachers, providing the children with some exercise equipment, training the canteen food handlers to prepare a healthy menu.	PA score (PAQ-C) in the intervention group increased by 0.37 from baseline to post-intervention and increased by 0.18 from baseline to 3-month post-intervention, while there was no significant change in the control group.
Hidrus et al., 2020	*n* = 70 Malaysia Adults M/F Type 2 diabetes	Brain breaks, 10-min exercise videos were uploaded to a WhatsApp group for the individuals in the intervention group to complete for 4 months.	The intervention group displayed a higher mean total PA compared to the control group across all time points (IPAQ-M).
Cheah et al., 2019	*n* = 155 Malaysia Adults M/F	Intervention groups were assigned weekly 90-min aerobic exercise sessions for 6 months.	No significant findings with regards to PA data.
Nik Yahya et al., 2019	*n* = 52 Indonesia Adults M/F	Intervention groups were assigned 3 times per week of 25-min aerobic exercise sessions for 10 weeks.	Significant increase in total PA (IPAQ) with intervention. Pre-intervention = 811.00 (648.50). Post-intervention = 1431.00 (459.00). No significant increase in the control group.
Pengpid et al., 2019	*n* = 375 Thailand Adults M/F Pre-hypertension, pre-diabetes	The intervention group underwent 6 group lifestyle counselling sessions over a period of 6 months.	No significant findings with regards to PA data.
Pichayapinyo et al., 2019	*n* = 35 Thailand Adults M/F Type 2 diabetes	Participants attended a 1-h group diabetes education session and received weekly 5 to 10-min interactive voice response calls for 12 weeks.	Pre-post changes in PA were significant with a mean increase of 0.7 (1.3) points out of 6 on the L-Cat scale.
Rizal et al., 2019	*n* = 322 Malaysia Children M/F	Brain breaks, exercise videos accumulating to a weekly total of 30 min were shown to students for 12 weeks.	No significant findings with regards to PA data.
Zulkifi et al., 2019	*n* = 65 Malaysia University students M/F	The 7-week intervention involved a student-centred approach and alternative assessments to evaluate students’ learning, focusing on accentuating participants’ roles during learning and assessing their knowledge and self-efficacy related to health education using alternative assessments.	Weekly recorded pedometer steps increased from 59,560 in week one to 87,286 in week seven, but no statistical test was done to determine significance.
Liau et al., 2018	*n* = 85 Singapore Adults M/F	The intervention group was subjected to two sets of self-regulation strategies: self-regulation strategy of mental contrasting with implementation intentions and self-monitoring for 3 weeks.	For overall PA (steps per week), there was an increase in PA, only for men, from 7124.37 to 9180.44 steps per week and no change for the control group. For final-week PA (steps per week), there was an increase in PA, only for men, from 7124.37 to 9002.84 steps per week, and a decrease for the men in the control group from M = 8679.42 to 7769.86.
Mok et al., 2018	*n* = 55 Malaysia Children M/F	An intervention involving nutrition education classes on healthy eating and active lifestyle; physical activity sessions; and active involvement of parents and teachers was conducted for 12 weeks.	There was a significant increase in PA levels (PAQ-C) from baseline to 15 months post-intervention, from 2.46 to 2.87.
Nguyen et al., 2018	*n* = 157 Vietnam Adults M/F	Participants were subjected to either storytelling or didactic intervention for 12 months.	Low PA prevalence (<600 MET-min) decreased more in the storytelling (17.2%) than in the didactic intervention group (9.8%). However, there was no indication if there was a statistical test done to determine significance.
Nur Emilia et al., 2018	*n* = 62 Malaysia Adults M/F	The intervention group had monthly meetings for 3 months to go through their log diaries and were given the motivation to increase their physical activity.	Pedometer step count between intervention and control group based on time: 1. Month 2: Mean difference (Control-Intervention) = −1165.00 [−2293.23, −36.76]. 2. Month 3: Mean difference (Control-Intervention) = −1619.00 [−2585.23, −652.76].
Omar et al., 2018	*n* = 178 Malaysia Adults F-only	The intervention group were encouraged to perform a physical activity package comprising: 1. 15-min daily brisk walking, 2. 30-min daily pillow dumbbell exercise, 3. Physical activity diary for self-monitoring, for 6 months.	No significant findings with regard to the PA data.
Suttanon et al., 2018	*n* = 277 Thailand Older adults M/F	Participants in the intervention group were provided with a 4-month multifactorial falls prevention programme, with a focus on balance training exercises and had handrails installed in their homes, or given walking assistive devices.	Increase in exercise frequency but not modified PASE score or total exercise time in the intervention group.
Widyastuti et al., 2018	*n* = 40 Indonesia Adults M/F	Participants in the control group were tasked to walk at the fastest pace possible at home for at least 30-min daily for 6 weeks, while participants in the intervention group were given 3 weekly 30-min sessions of supervised standard exercise training for 6 weeks in addition to the above.	No significant between-group differences.
Yatim et al., 2018	*n* = 45 Malaysia Adults M/F Hypertension	Participants were given a self-management guidebook, covering topics and hands-on self-management activities related to hypertension (i.e., living with hypertension, healthy eating and hypertension, physical activity for hypertensive patients, and know your medicines) for 4 weeks.	The number of days the participants spent on vigorous physical activity significantly increased from 0.56 to 1.81 (d/wk) between baseline and post-1 week, while walking time significantly. decreased from 33.33 to 23.33 (min/d) between baseline and post-1 month.
Chawla et al., 2017	*n* = 490 Thailand Children M/F	The participants were subjected to a multicomponent healthy lifestyle program that focused on the promotion of healthy eating and being physically active for 6 months.	No significant findings with regards to PA data.
Huei et al., 2017	*n* = 189 Malaysia Adults M/F	Participants were split into 1 control and 2 intervention groups for 16 weeks. The first intervention group was subjected to point-of-decision prompts to motivate them to walk more. The second intervention group was given a weekly 1-h aerobics class.	Only significant differences between the step count of the aerobics and control group but no indication of which time points the statistical significance applied to.
Juliana et al., 2017	*n* = 31 Malaysia Adults F-only	The intervention group was given a package of a variety of diet and physical activity guidelines for 16 weeks.	Significant effect of intervention in the intervention group, when compared to control, for PA in min/wk (3171 at baseline to 3355 at 16 weeks) and PA in activity score (7457 at baseline to 8298 at 16 weeks) using Short Questionnaires to Assess Health-Enhancing Physical Activity (SQUASH).
Karintrakul et al., 2017	*n* = 45 Thailand Adults F-only	The intervention group received nutrition counselling. The study mentions three, 30 to 45-min counselling sessions, followed by 5–10 min between sessions over a total of 12 weeks.	No significant findings with regards to PA data.
Sanee et al., 2017	*n* = 100 Thailand Adults F-only	Participants were subjected to a two-part intervention for a total of 17 weeks, the first consisting of a peer leader training program and the second consisting of a peer leader-led program. The program consisted of whole-group and small-group peer support and discussion. Personalised goals were set, education for healthy eating habits and PA for weight management were disseminated, as well as addressing personal barriers to change, maintaining motivation and encouraging them to use the additional material available to them.	No significant findings with regards to PA data.
Tran et al., 2017	*n* = 337 Vietnam Adults M/F	The intervention included four educational sessions, a booklet, a resistance band and walking groups for 6 months.	Significantly greater moderate activity (61 vs. 30), walking time (588.3 vs. 326.7 min/wk) and total PA (862.7 vs. 502.9 min/week) in the intervention compared to the control group post-intervention using IPAQ.
Zahtamal et al., 2017	*n* = 34 Indonesia Adults M/F	One group received a multilevel educational intervention that targeted various levels of the individual’s social support system, while the other group only received health education at an individual level for 12 weeks.	No significant findings with regards to PA data.
Finkelstein et al., 2016	*n* = 800 Singapore Adults M/F	There were 4 intervention groups: a control group, a group that was only given a Fitbit pedometer, a Fitbit and charity incentive, as well as a Fitbit and cash incentive group. Participants in the non-control groups were asked to complete a certain number of steps per week for 6 months.	After 12 months: - Fitbit group had significantly higher MVPA bout min per week than the control group in the full (37), insufficiently active (24) and active (68) samples. - Charity group had significantly higher MVPA bout min per week than the control group in the full (32), insufficiently active (25) and active (49) samples. - Cash group had significantly lower MVPA bout min per week than the Fitbit group in the full (−23) and active (−42) samples. - Fitbit group had significantly higher mean daily steps than the control group in the active (980) sample. - Cash group had significantly higher mean daily steps than the control group in the full (500) and active (960) samples.
Haya et al., 2016	*n* = 48 Indonesia Adults F-only	Individuals in the intervention group were given maternal health education 6 times for 12 weeks (60 min per class period), using a participation discussion method and booklets containing practical guides on childhood obesity management. Mothers in the control group received health education only once for 60 min at the beginning of the study.	Significant increase in PA level in the intervention group (0.04) and a significant decrease in PA level in the control group (−0.01).
Ibrahim et al., 2016	*n* = 268 Malaysia Adults M/F Pre-diabetes	Participants in the intervention group received twelve group-based sessions of 90 min each and a minimum of two individual counselling sessions with the dietician and researcher to reinforce behavioural change, for 12 months.	Significantly higher PA (MET-min/wk) in the intervention group than in the control group at 6-month (66.5) and 12-month (183.2) measures.
Jafar et al., 2016	*n* = 100 Singapore Adults M/F Hypertension	The interventions were related to training the *physicians* in the treatment program algorithm, subsidy for antihypertensive medication, training nurses in motivational conversation and structured follow-up over the telephone over 8 weeks.	No significant findings with regards to PA data.
Müller-Riemenschneider et al., 2016	*n* = 43 Malaysia Older adults M/F	Participants in the intervention group were sent 60 SMS text messages over the course of 12 weeks. These messages provided instructions for exercise and provided praise/rewards for efforts towards exercise behaviour.	No significant findings with regards to PA data.
Nhung et al., 2016	*n* = 60 Vietnam Adults F-only Post-menopause	Participants in the intervention group received 6 capsules containing acylated steryl glucosides, while the placebo group took 6 placebo isocaloric capsules over 6 months.	No significant between-group differences.
Ethisan et al., 2015	*n* = 102 Thailand Adults M/F	Participants in the intervention group received a Group-Mediated Lifestyle Physical Activity program for 6 months. This included group-based PA, group-mediated education and home-based PA.	Mean score of health benefits from physical activity increased from 23.2 to 40.7 in the intervention group, while it decreased from 20.7 to 4.6 in the control group.
Huynh et al., 2015	*n* = 199 Philippines Children M/F	Parents received three sessions of dietary counselling administered at baseline, weeks 4 and 8 post-baseline, while children received two servings of oral nutrition supplement per day for 48 weeks.	Parent-reported children’s PA levels increased from 7.9 to 9.0 from baseline to 48 weeks (VAS).
Ng et al., 2015	*n* = 246 Singapore Older adults M/F	The participants were split into 1 control and 4 intervention groups, including nutritional, cognitive training, physical training and a combined interventions group over 12 months.	Mean change from baseline (average time, min/d): 6 months × Nutritional = 96.2 [57.8, 134.7]. 12 months × Nutritional = 110.1 [71.9, 148.2].
Sazlina et al., 2015	*n* = 69 Malaysia Older adults M/F	Participants were split into personalised feedback (PF) about physical activity patterns group, peer support (PS) group and a control group. Both PF and PS groups received structured personalised feedback and usual diabetes care. Participants in the PS group received support from peer mentors in addition to the above. This was done over a period of 12 weeks.	The PS group demonstrated significantly greater mean daily pedometer readings compared to the PF group at weeks 12 (1416 steps/d) and 36 (1416 steps/d) and were significantly greater compared to the control group at weeks 12 (2265 steps/d), 24 (2586 steps/d) and 36 (2084 steps/d).
Ngo et al., 2014	*n* = 285 Singapore Children M/F	The intervention comprised targeted education on myopia and good eye care habits, structured weekend outdoor activities and incentives for children to increase their daily steps, as measured via pedometers (step counters). The control group only received resources on myopia prevention and the health benefits of physical activity. The intervention lasted 9 months.	The intervention group reported higher outdoor time across the whole week (14.75 vs. 12.4 h/wk) and on the weekend (2.89 vs. 2.4 h/d) compared to the control group during a 6-month interim measure. However, there were no significant differences between both groups at the end of the entire intervention.
Sriramatr et al., 2014	*n* = 220 Thailand Adults F-only	Participants were randomly allocated into 4 groups for 3 months: intervention with/out pre-test and control with/out pre-test. The intervention groups were subjected to an internet-based program where they recorded their average physical activity, set physical activity goals for the next week.	With pre-test: The intervention group recorded more steps/d than those in the control (11,654 vs. 8194 steps/d) and leisure time activity score (54.14 vs. 37.58). Without pre-test: The intervention group recorded more steps/d than those in the control (10,601 vs. 7570 steps/d) and leisure time activity sore (59.50 vs. 39.32).
Suwanpasu et al., 2014	*n* = 46 Thailand Older adults M/F	Participants in the intervention group were subjected to a physical activity enhancing program with a physical training component and efficacy-based intervention. The total duration of the intervention was not stated.	Significantly higher post-test measures of PA in the intervention group compared to the control group (961.37 MET-min/wk).
Finkelstein et al., 2013	*n* = 285 Singapore Children M/F	The intervention group received information on structured weekend outdoor activities and pedometer step programs for 3 months. Families were encouraged to attend sessions at least twice a month.	At follow-up, the intervention group had significantly higher pedometer steps than the control group across the entire week (958), weekdays (848) and weekends (1239).
Lua et al., 2013	*n* = 380 Malaysia University students M/F	The intervention programme employed was developed based on the latest Malaysian dietary guidelines. All included messages were delivered through 3 modes: conventional lectures, brochures and text messaging. The intervention lasted 10 weeks.	After 10 weeks, the intervention group had higher MET-min/wk than the control group across walking (764.2), moderate activity (333.4), vigorous activity (413.4) and total (1548.8).
Soon et al., 2013	*n* = 56 Malaysia Adults M/F	The intervention group was subjected to a combined physical activity and dietary intervention for 12 weeks. The intervention activities included lectures and group discussion sessions.	No significant findings with regards to PA.
Nguyen et al., 2012	*n* = 4650 Vietnam Adults M/F	In the communes selected for intervention, a hypertension management programme was implemented and integrated with the primary health care system. The intervention lasted 3 years.	Physical inactivity levels *increased* in the intervention group after 3 years (6.8% to 12.8%).
Tan et al., 2011	*n* = 164 Malaysia Adults M/F Type 2 diabetes	An education intervention was carried out consisting of 3 monthly sessions across 3 months, addressing the self-care practices of healthy eating, being active, medication adherence. and self-monitoring of blood glucose (SMBG). The 2nd and 3rd sessions centred around the SMBG results, exploring problem-solving skills related to hyperglycaemia, hypoglycaemia, sick day and emotional episodes.	After 12 weeks, total PA was higher in the intervention group (15.50) than the control group (12.73), but no difference within-group for either group (Revised Diabetes Self-care Activities Questionnaires modified from the Diabetes Self-care Activities Questionnaire).
Wafa et al., 2011	*n* = 107 Malaysia Children M/F	The intervention group was subjected to a parent-centric intervention focused on changing the behaviours to treat childhood obesity. There was a total of 8 intervention sessions, over 26 weeks, directed at the parents and participating children attended a physical activity session led by an exercise instructor.	No significant findings with regards to PA.
Chia, 2009	*n* = 490 Singapore Children M/F	The programme involved a 10-week infusion of daily physical play between 20 to 45 min during school curriculum hours, either as stand-alone additional play sessions or as part of an extended recess, where pupils could have light refreshments and play.	One school had an increase in the number of within-school-hours steps from 3742 to 4642, while another school had an increase from 4520 to 4984.
Muda et al., 2006	*n* = 91 Malaysia Adults M-only	The intervention group was given individual counselling on physical activity by the main researcher based on patient-centred assessment and counselling for exercise and monthly aerobic exercise. Group education was given at month 3, followed by monthly phone calls for the last 3 months.	Total energy expenditure was higher in the intervention group (3.08 kcal/kg/d) compared to the control group (0.38 kcal/kg/d) at the end of the 6-month intervention (7-day Physical Activity Recall).
Hadju et al., 1998	*n* = 129 Indonesia Children M-only	Individuals in the intervention group were subjected to albendazole injections for 6 months.	After 6 months, the activity increase in the intervention group (~0.3 METs) was higher than in the control group. Only graphical representations were provided.

^1^ M refers to male; F refers to female, ^2^ Numbers in (round brackets) show the standard deviation; numbers in [square brackets] show the confidence intervals; this is different across studies depending on what information they provide.

## Data Availability

Data is contained within the article.
